# How a High-Gradient Magnetic Field Could Affect Cell Life

**DOI:** 10.1038/srep37407

**Published:** 2016-11-18

**Authors:** Vitalii Zablotskii, Tatyana Polyakova, Oleg Lunov, Alexandr Dejneka

**Affiliations:** 1 Department of Optical and Biophysical Systems, Institute of Physics of the Academy of Sciences of the Czech Republic, Prague, 18221, Czech Republic

## Abstract

The biological effects of high-gradient magnetic fields (HGMFs) have steadily gained the increased attention of researchers from different disciplines, such as cell biology, cell therapy, targeted stem cell delivery and nanomedicine. We present a theoretical framework towards a fundamental understanding of the effects of HGMFs on intracellular processes, highlighting new directions for the study of living cell machinery: changing the probability of ion-channel on/off switching events by membrane magneto-mechanical stress, suppression of cell growth by magnetic pressure, magnetically induced cell division and cell reprograming, and forced migration of membrane receptor proteins. By deriving a generalized form for the Nernst equation, we find that a relatively small magnetic field (approximately 1 T) with a large gradient (up to 1 GT/m) can significantly change the membrane potential of the cell and thus have a significant impact on not only the properties and biological functionality of cells but also cell fate.

In recent decades, the interaction of magnetic fields with living cells and organisms has captivated the interest of a broad scientific community drawn from a wide spectrum of disciplines, including biology, physics, chemistry, medicine and nanotechnologies. Extensive progress in experimental techniques and the design of new magnetic materials has resulted in the burgeoning development of new approaches to reveal the targets of magnetic fields on the intracellular and molecular levels^1^,^2^,^3^.

The scientific literature is filled with thousands of works on the responses of living organisms to low, moderate and strong magnetic fields, for review see^4^,^5^,^6^,^7^,^8^,^9^,^10^. However, the biological effects related to the gradient of the magnetic fields are poorly discussed. Relatively few studies have quantified magnetic gradient actions at the intracellular level. Nevertheless, namely spatially non-uniform magnetic fields with a large enough gradient are capable of significantly altering cell functions and even organisms. For example, a large-gradient magnetic field can affect FLG29.1 cell differentiation to form osteoclast-like cells^11^. Under HGMFs, significant morphologic changes in osteoblast-like cells occurred, including expansion of the endoplasmic reticulum and mitochondria, an increased number of lysosomes, distorted microvilli, and aggregates of actin filaments^12^. The early embryonic growth of the leopard frog (*Rana pipiens*) was strongly inhibited by a 1 T magnetic field with a high gradient of 84 Tm^−1 ^13.

When analyzing effects of magnetic fields on living cells, tissue and organisms, one should keep in mind that in most cases, the biological cells and tissue are diamagnetic with susceptibility very close to that of water^14^. Therefore, the differences in the diamagnetic susceptibilities of cellular components are very low, which leads to tiny effects. In contrast, the exposure of cells and organisms to high-gradient magnetic fields (HGMFs) reveals many intriguing effects that might be directly related to the magnetic gradient force exerted on the whole cell and its organelles. Indeed, the magnetic force acting on a magnetic dipole moment is proportional to the field gradient, i.e., *F* *∝ ∇B* (where *B* is magnetic induction). In the case of cells suspended in a weakly diamagnetic medium, the volumetric force is *F* *∝ ∇B*^*2*^. Thus, after achieving a sufficient magnetic gradient, significant changes in cell functions, shape and spatial organization might be possible. In spite of the many interesting effects related to the application of spatially non-uniform magnetic fields, a key problem—how high-gradient magnetic fields change cell machinery—has never been carefully examined. Special interest exists in the case when the applied magnetic field dramatically changes in value and direction across the cell body. Here, the important question is: how will the cell respond and adapt itself to a high magnetic field gradient? From point of view of physics, the answer is the following. Considering the cell as a droplet of diamagnetic liquid placed in a non-uniform magnetic field, one can conclude that such a droplet will divide itself into several smaller drops to satisfy the minimum of the total system energy. A qualitatively similar effect—ferrofluid droplet division in a non-uniform magnetic field (B = 68 mT) with gradient, dB/dz = 6.6 Tm^−1^—was recently reported in^15^. It is obvious that living cell mechanics is much more complex than that of a liquid droplet. Nevertheless, in spite of the small contribution of diamagnetic forces in the interplay between biological and physical factors in the cell machinery, the role of the magnetic gradient force can increase with increasing magnetic gradient. There are no principal physical limitations the increase of magnetic field gradients. For example, micro-magnet arrays can produce magnetic fields that are spatially modulated on the micron scale with a gradient up to 10^6^ Tm^−1^ at micro-magnet edges^16^,^17^,^18^,^19^,^20^. In the vicinity of a magnetic nanostructure, magnetic field gradients can be large enough (up to 10^7^ Tm^−1^) for the field to vary appreciably over the separation between electrons in a radical pair^21^ thereby modulating the intracellular magnetocatalytic activity. Moreover, theoretical results^22^ show that an HGMF can lead to a significant enhancement of the performance of a chemical biocompass believed to exist in certain animals and birds. A non-uniform magnetic field up to 610 T with a gradient on the order of 10^6^ Tm^−1^ on the millimeter scale was recently generated with a laser-driven capacitor-coil target by proton deflectometry^23^.

To identify the intracellular targets and molecular effectors of magnetic fields and to reveal the underlying mechanisms, many complex multidisciplinary problems must be solved. As is often the case when multiple disciplines address a complex scientific problem, theoretical models and mathematical equations can provide a unifying platform to synergize the efforts. We present a theoretical framework for a fundamental understanding of the effects of magnetic gradient forces on intracellular processes, highlighting new directions of the study of living cell machinery affected by magneto-mechanical forces.

## Results

### Direct influence of a high-gradient magnetic field on the resting membrane potential of a cell

Membrane voltage is a key parameter regulating cell properties, machinery and communication. In general, electricity and the interaction of electric charges play major roles in the life of a cell. Indeed, a simple estimation (see Methods) of the electrostatic energy stored in the membrane of a spherical cell with radius 10 μm and membrane voltage 70 mV is E ≈ 10^−14^–10^−13^ J, which is 6–7 orders of magnitude larger than thermal fluctuation energy and much larger than the energies of chemical bonds and membrane bending^24^, which determine many membrane-mediated intracellular processes, such as shaping, rigidity, endocytosis, adhesion, crawling, division and apoptosis. Thus, the electrostatic contribution of the bending energy of charged cell membranes is large enough^25^, and in a first approximation, the cell membrane rigidity is proportional to the square of the membrane voltage. Qualitative analysis presented in^26^,^27^ shows that cells (able to proliferate rapidly, undifferentiated) with low values of membrane potential, which tend to depolarized, are highly plastic. In contrast, cells that are mature, terminally differentiated, and quiescent tend to be hyperpolarized. It should be stressed here that the membrane potential is not simply a reflection of the cell state but a parameter allowing the control of the cell fate, for example, artificial depolarization can prevent stem-cell differentiation, whereas artificial hyperpolarization can induce differentiation. Below, we analytically analyze the possibility of driving the membrane potential with externally applied, high-gradient magnetic fields.

When a high-gradient magnetic field is applied to a cell in medium, the magnetic gradient force acts on ions and can either assists or oppose ion movement through the membrane. The magnetic gradient force is given by 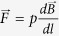, where *p* is the magnetic dipole moment of the ion, ***B*** is the magnetic induction, and the derivative is taken with respect to direction ***l***, which is parallel to the magnetic dipole moment of an ion, ***l***//***p***. Bearing in mind the former expression for the magnetic gradient force, in this case, when the ions diffuse in the presence of an HGMF, the Nernst equation reads as (see Methods)


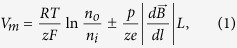


where *e* is the electron charge, *z* is the ion valence (z = +1 for a positive, univalent ion), *F* is the Faraday constant, *R* is the gas constant, *T* is the absolute temperature, *V*_*m*_ is the potential difference between the two membrane sides, and *n*_*o*_ and *n*_*i*_ are the ion concentrations outside and inside a cell, *L* is the half-cell size. On the right side of Eq. 1, the second term describes the magnetic contribution to the resting potential. Thus, Eq. 1 represents a generalized form of the Nernst equation derived with regard to the influence of a high-gradient magnetic field. Depending on the direction of the magnetic gradient (“+” or “−” in Equation 1), an HGMF can cause either membrane potential depolarization or hyperpolarization, which regulates not only the entry of sodium, potassium, and calcium ions and biologically relevant molecules to the cell but many pivotal cell characteristics and functions. The key question is how large the gradient value should be to achieve a direct effect of the magnetic fields on the membrane potential. To address this question, we estimate the contribution of the magnetic term to the equilibrium membrane potential given by Eq. 1. For this estimation, the values of the magnetic moments of ions that create the membrane potential should be known. Typical ion-channel species (K^+^, Ca^2+^, Na^+^) and nearby water molecules are electron spin paired, so they have no spin electron magnetic moment and their magnetic moment is due to nuclear spin. It is interesting that ^40^Ca^2+^ ions have no nuclear magnetic moment. The magnetic moments of these ions are very small and are on the same order of magnitude as the nuclear magneton, μ_n_ = 5.05 10^−27^ J/T: *p*_*Na+*_ = 2.22μ_n_ (sodium-23), *p*_*K+*_ = 0.39μ_n_ (potassium-39), *p*_*Cl*−_ = 0.821μ_n_ (chloride-35), and *p*_*Ca2+*_ = 0 (calcium-40). Among these ions, Na^+^ has the largest magnetic moment and Ca^2+^ has zero electronic and nuclear magnetic moments. For comparison, we list the magnetic moment values of relevant molecules: for H_2_0 (para, antiparallel nuclear spins) *p* = 0 and H_2_0 (ortho, parallel nuclear spins) *p* = μ_n_ and for hemoglobin Fe^2+^, the average magnetic moment measured for whole blood is equal to 5.46 μ_B_/Heme^28^ (where μ_B_ is the Bohr magneton, μ_B_/μ_n_ ≈ 1836). Due to the nuclear spins of the hydrogen atoms, water consists of a mixture of spin zero (para) and spin one (ortho) molecules. The equilibrium ratio of *ortho* to *para* molecules is 3∶1^29^, making 75% of water molecules magnetically active in sufficiently strong magnetic fields. HGMF, due to the relatively large magnetic moments of Na^+^ ions, can affect the formation of the action potential of a nerve cell. By estimation of the magnetic addition in Eq. 1 for the above values of magnetic moments of K^+^ and Na^+^ ions and biologically relevant molecules to the cell, we find that an externally applied magnetic field with a gradient value on the order of 10^8^–10^9^ Tm^−1^ can directly change the cell membrane potential by 1–10 mV. For example, in neuron cells, the opening of Na^+^ and K^+^ voltage-gated ion channels occurs with membrane potential depolarization as small as 7–12 mV^30^. In this case, the direct effect of the application of HGMFs to the cell can manifest itself through the change of the probability of opening/closing the voltage-gated ion channels. However, as estimated above, to achieve membrane potential depolarization or hyperpolarization, one has to apply an HGMF with a gradient on the order of 10^9^ Tm^−1^. The possibility of achieving such high values of magnetic gradient is described in the next section.

The currently reachable magnetic gradient (up to 10^6^–10^7^ Tm^−1 ^23,31) has indirect effects related to the application of HGMGs to cells. First, the effects of magnetic fields with a gradient on the order of 10^6^ Tm^−1^ can manifest itself through the change of the probability of opening/closing mechanosensitive ion channels. On the other hand, mechanical stress in the cell membrane can directly drive ion channel gating^32^,^33^,^34^. Moreover, the membrane potential can be changed through agitation of the membrane ion channels. Recent studies have demonstrated the importance of the membrane potential value in the regulation of cell functions and signaling at the multicellular level^33^, especially in relation to ion channel activity. For example, cancer cells tend to have low membrane potential (in absolute value), which has been connected to the overexpression of specific ion channels^35^. Highly differentiated tumor cells (human hepatocellular carcinomas: Tong, HepG2, Hep3B, PLC/PRF/5, Mahlavu, and HA22T) have paradoxically small membrane potentials^36^. The membrane potential controls the adipogenic and osteogenic differentiation of stem cells^37^, which suggests the possibility to drive the differentiation pathway. The membrane potential plays a key role in the spatial organization of cytoskeletal and cell division-related proteins, mainly influencing bacterial cell division^38^.

Static homogeneous magnetic fields can also affect the diffusion of biological particles through the Lorentz force and hypothetically change the membrane potential. However, the results presented in^39^ show that in solution, the Lorentz force can suppress the diffusion of univalent ions (e.g., Na^+^, K^+^, and Cl^−^), but the threshold magnetic field is extremely high, approximately 5.7 · 10^6^ T (which is 2–4 orders of magnitude less than the magnetic field at a magnetar). On the other hand, the theoretically predicted threshold of gradient fields for producing a change in ion diffusion through the magnetic gradient stress is more than 10^5^ T^2^m^−1^ for paramagnetic molecules FeCl_3_ and 0_2_ and plasma proteins^39^. Thus, in low and moderate magnetic fields, the biological effects should be rather dependent on the magnitude of the magnetic field gradient and not on the strength of the magnetic field, as was recently demonstrated in experiments with THP-1 cells^32^. The magnetic systems capable of generating HGMFs and formulas allowing rapid estimation of the magnetic field gradient are described in Methods and Table 1. We now consider possible applications of these magnetic systems to control cell functions.

### Effects of an HGMF through intracellular mechanical stress

A possible alternative mechanism of cell response to HGMFs relies on the fact that magneto-mechanical stress can affect mechanosensitive membrane ion channels, for example, TREK-1 ion channels, which are stretch-activated potassium channels^40^,^41^. It is believed that a cell may have 10^2^–10^4^ ion channels, and the probability of any of them being open (at any given time) is typically in the range of a few to a few tens of percent^42^,^43^. Magnetic gradient forces exerted on cells impose mechanical stress on the plasma membrane and cell body. The cell senses this stress and elicits a mechanoelectric transduction cascade that initiates a response. In the cell membrane, mechanosensitive ion channels are responsible for transducing mechanical signals into electrical signals. Additional membrane tension, in our case induced by the high-gradient magnetic field, can increase the probability of mechanosensitive channel opening^44^. Thus, plasma membrane mechanical stress activates transient receptor potential (TRP) channels^45^. Below, we calculate the mechanical forces and stress in a cell placed in an HGMF.

The volume density of the magnetic gradient force (in Nm^−3^) acting on a cell is


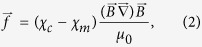


where χ_m_ is the susceptibility of the medium, χ_c_ is the susceptibility of the cell, and μ_0_ is the vacuum permeability. In Eq. 2, the difference of susceptibilities, Δχ = χ_m_ − χ_c,_ defines the magnetic force direction: attraction or repulsion of a cell to/from the area with a high-gradient magnetic field. This force causes mechanical stress in the whole cell and cell membrane. Analysis of the possible biological effects of the action of magnetic gradient forces with volume density given by Eq. 2; one can compare these forces with the gravitational force density, *f*_*g*_ = *ρg* = 10^4^ Nm^−3^ (where *ρ* is the density of water and *g* is the acceleration of gravity). Assuming Δχ to be 10–20%^46^ of the diamagnetic susceptibility of water (χ_w_ = −9 ⋅10^−6^ in SI), B = 1 T and |∇B| = 10^6^ Tm^−1^, from Eq. 2, we obtain the magnetic force density *f* = (0.7–1.4) · 10^6^ Nm^−3^, which yields *f* ≫ *f*_*g*_. Because the gravitation force (microgravity) or weightlessness (e.g., by magnetic levitation) affect cell development, growth and functions^47^,^48^, significant effects of the magnetic gradient forces would be expected. For example, the applied magnetic fields with gradient of approximately ∇B^2^ ≈ 10^3^ T^2^m^−1^ were shown to change the subcellular morphology of osteoblast-like cells^12^, and diamagnetic levitation plays a major role in the observed effects. Thus, significant effects on cell machinery caused by the magnetic gradient forces are expected. The magnetic forces that are exerted on the cell body are transmitted to the cell cytoskeleton and cell membrane. Even tiny mechanical forces that are slightly larger than the thermal fluctuation forces of less 1 pN (see Methods) can significantly affect cell functionality^32^,^49^,^50^,^51^.

The magnetic gradient forces given by Eq. 2 can directly drive paramagnetic cells and molecules. In general, cells are diamagnetic. However, recent research shows the existence of nonerythroid cell lines derived from human cell cancers that are sufficiently paramagnetic^52^. Their paramagnetic behavior makes it possible to affect cell motion by application of an HGMF. Moreover, intracellular and intercellular free radicals, such as O_3_, NO, and NO_2_ and molecules FeCl_3_ and O_2_, are also paramagnetic and can be redistributed by both the Lorentz force and magnetic gradient force, as known from electrochemistry^53^,^54^.

**Figure 1:**
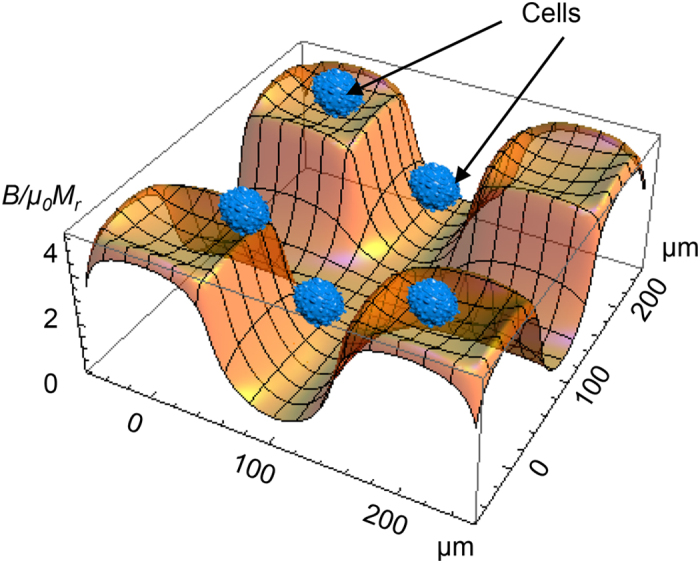
Spatial distribution of the scaled modulus of the magnetic field (*B/μ*_*0*_*M*_*r*_) calculated in the plane 5 μm above four micromagnets (*M*_*r*_ is remanent magnetization). Several cells are schematically drawn to demonstrate that the magnetic field varies in the same length scale as the cell mean size. The micromagnet sizes are 100 × 100 μm, and the spacing is 100 μm.

**Figure 2:**
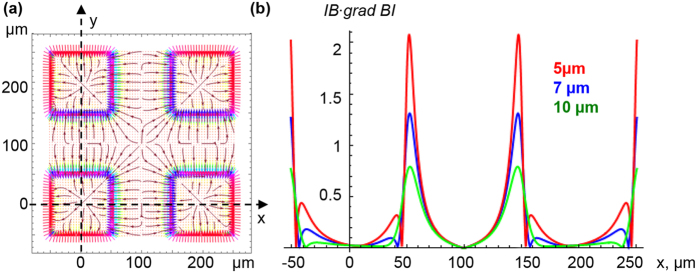
Spatial distribution of the scaled planar component of the magnetic gradient (**a**) 5 μm above the micromagnets shown in Fig. 1. (**a**) Vector field {∇_x_(*B/μ*_*0*_*M*_*r*_)^2^,∇_y_(*B/μ*_*0*_*M*_*r*_)^2^ } multiplied by the micro-magnet size. Arrows indicate the directions of the magnetic gradient forces. (**b**) Scaled modulus of the planar magnetic gradient (∇_x,y_(*B/μ*_*0*_*M*_*r*_)^2^) multiplied by the micro-magnet size as a function of the *x*-coordinate. The gradient values were calculated along the *OX*-axis at distances from the magnet tops: 5 μm, 7 and 10 μm.

One of the key functions of cells is ordering in space and time. High-precision cell positioning with micromagnets is a promising approach for tissue engineering^20^. Indeed, the magnetic gradient force (Equation 2) is capable assisting cell migration to areas with the highest magnetic field gradient. It was recently demonstrated in ref. 46 that micromagnet arrays (with lateral size of 30–50 μm and the same neighboring distances) coated with parylene produce high magnetic field gradients (up to 10^6^ Tm^−1^) that affect cell behavior in two main ways: i) causing cell migration and adherence to a covered magnetic surface and ii) elongating the cells in the direction parallel to the edges of the micromagnet. The results of the calculations of the magnetic field and gradient distributions above four micromagnets are shown in Figs 1 and 2. The field and magnetic-gradient force distributions were calculated analytically using explicit expressions for the magnetic stray fields^55^. As seen from Figs 1 and 2, there are several areas with the highest magnetic gradient. Thus, in the experiments^46^, driven by magnetic gradient forces (Equation 2), cell migration was observed towards the areas with the strongest magnetic field gradient, thereby allowing the buildup of tunable, interconnected, stem cell networks.

Recent studies indicate the crucial influence of external mechanical and magnetic forces on the cell shape, function and fate through physical interactions with the cytoskeleton network^46^,^49^,^56^.

### Local change of membrane potential and lateral migration of membrane receptor proteins in the vicinity of magnetic nanoparticles

**Figure 3:**
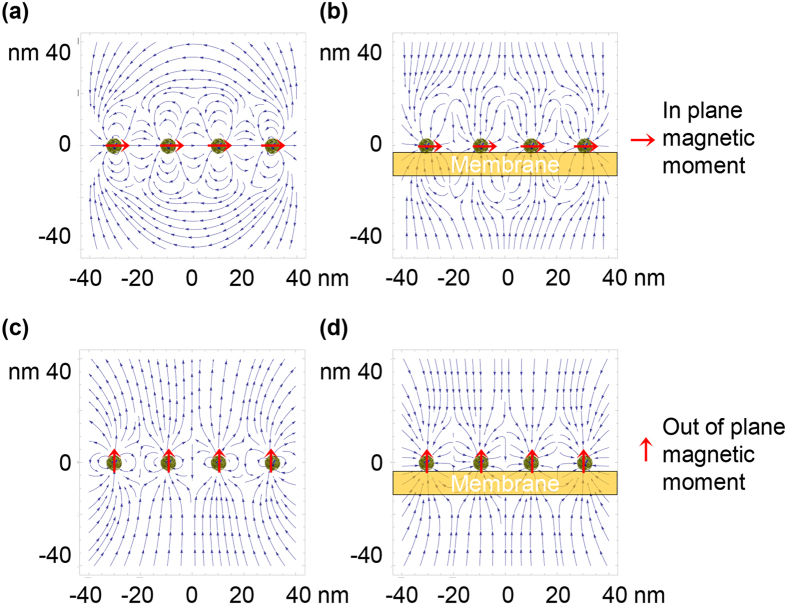
Vector fields of the magnetic induction (**a** and **c**) and magnetic gradient (**b** and **d**) in the vicinity of four magnetic nanoparticles magnetized parallel and perpendicular to the membrane surface. In (**b** and **d**) arrows indicate the directions of the magnetic gradient forces.

A chain of magnetic nanoparticles (MNPs) placed on a cell membrane can create spatially modulated magnetic flux distributions with a sufficient gradient. The magnetic gradient forces localized near the MNPs affect cell functions in two main ways: i) changing the resting membrane potential, as predicted by Eq. 1, and ii) generating local magnetic pressure that can cause membrane deformation, resulting in cell membrane blebbing. The former can occur locally as a consequence of a very high field gradient, as given by Eq. 15 (Methods). For magnetite (Fe_3_O_4_) MNPs with *M*_*s*_ = 510 kAm^−1^ and *R* = 5 nm, estimation based on Eq. 15 gives |∇B_r_| ≈ 2.6 10^8^ Tm^−1^ at the membrane surface. This gradient magnitude is enough to change the resting potential by a few mV even though the ions driving the membrane potential have only nuclear values of magnetic moments. The second is related to the magnetic pressure due to the difference of the magnetic susceptibilities of the lipid membrane and cytosol. In the vicinity of an MNP, the magnetic pressure at the cell membrane is *P*_*MNP*_* = fV/S = fh*, where *V* and *S* are the volume and areas of a small part of the membrane and *h* is the membrane thickness. The analytical expression for this pressure is given in Methods. For chains of MNPs with parallel and perpendicular orientation of the magnetic moments with respect to the membrane surface, the magnetic pressure (*P*_*MNP*_) acts in directions perpendicular and parallel to the membrane, as it illustrated in Figs 3 (a–d) for two chains consisting of four MNPs. The magnetic pressure causes an imbalance in the osmotic and hydrostatic pressures, which in turn changes the flux of ions transported through the cell membrane^32^. To estimate the magnetic pressure one should know the magnetic susceptibilities of the cellular contents, which can be found in ref. 57 and the references therein. In particular, the magnetic susceptibilities of proteins, lipids and water are χ_p_ = −9.726 10^−6^, χ_lip_ = −8.419 10^−6^ and χ_w_ = −9.035 10^−6^ (all in SI). Thus, proteins are more diamagnetic than water, i.e., χ_p_ < χ_w_. Lipids are less diamagnetic than proteins and water (χ_lip_ > χ_p_ and χ_lip_ > χ_w_), resulting in their “quasi-paramagnetic” behavior with respect to lipids and the cytosol. Due to the difference of the magnetic susceptibilities of proteins and lipids, the membrane receptor proteins are attracted to the area with the highest magnetic field gradient generated by MNPs (see Fig. 3). Estimations of the lateral magnetic pressure (Equation18, Methods) acting on the membrane receptor protein at *h* = 5 nm, *r* ≈ *R* = 5 nm, *M*_*s*_ = 510 kAm^−1^ (magnetite MNPs) and Δχ = χ_p_ − χ_lip_ = 1.3 10^−6^ result in P = 1.7 Pa. This pressure can force the lateral migration of membrane receptor protein towards the high-gradient field area. Moreover, cell membranes accommodate domains with heterogeneous sizes ranging from 10 to 200 nm, which are enriched in cholesterol and saturated lipids. Because the magnetic susceptibility of cholesterol is close to that of protein, χ_ch_ = −9.236 10^−6 ^57, these domains are subjected to the lateral magnetic pressure and forced diffusion occurs. This redistribution of the membrane domains can play a pivotal role in altering membrane functions.

### Magnetically assisted cell division

The first hint of the possibility of cell division by an HGMF was discussed above in relation to an experiment on the division of ferrofluid droplets in a moderate magnetic field with gradient dB/dz = 6.6 Tm^−1^. The diamagnetic susceptibility of a cell is much smaller than that of a ferrofluid droplet. When discussing the effects of HGMFs on cells, we consider at least six orders of magnitude larger field gradients. Because the magnetic gradient force is proportional to the product of the magnetic susceptibility and the field gradient (Equation 2), in our case, one can expect a similar effect, i.e., stimulation of cell division by magnetic gradient forces. Magnetic gradient forces can be significantly increased by loading cells with magnetic nanoparticles. In experiments described in ref. 58, localized, nanoparticle-mediated magnetic forces were applied to HeLa cells through a magnetic field with a gradient from 2.5∙10^3^ Tm^−1^ to 7∙10^4^ Tm^−1^. Under the largest gradient, the cells loaded with magnetic nanoparticles exhibited ‘pull-in’ instability. However, under lower magnetic gradients and lower intracellular mechanical stress, biasing of the metaphase plate during mitosis was observed, which indicates that in HGMFs, magneto-mechanical stress is able to assist in the division of cells free of magnetic nanoparticles.

**Figure 4:**
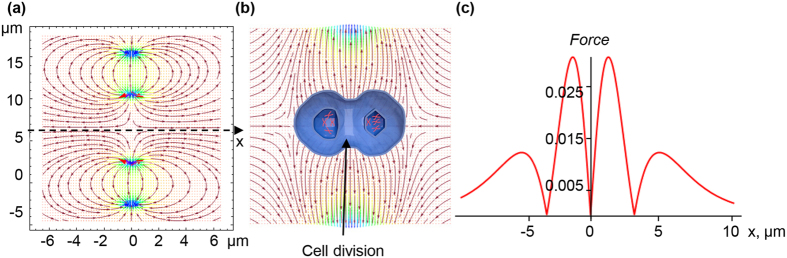
Vector fields of the magnetic induction (**a**) and magnetic gradient forces (**b**) between the two, pole-to-pole magnetic slabs and cell division. (**c**) Magnetic gradient forces (Equation 2) normalized to Δχ*a*^*−1*^*μ*_*0*_*M*_*r*_^2^ as a function of the *x*-coordinate. A hypothetical division of a cell in the highly non-uniform magnetic field (the central area) is illustrated.

Therefore, we hypothesize that cell division can be either induced or assisted by a specifically, spatially modulated, magnetic gradient field. An example of such a magnetic field configuration and magnetic gradient force distribution is shown in Fig. 4, illustrating the field and its gradient (normalized ∇B^2^) distributions generated in the gap between two uniformly magnetized magnets faced pole-to-pole. The field and gradient were calculated using the explicit analytical expressions for the magnetic field induction of rectangular, magnetized prisms^55^,^59^. Figure 4b shows that between the magnetic poles, on the left and right parts of the central area, the magnetic gradient forces have opposite directions. If the mean size of this area is comparable to the cell size, a cell placed here will be subjected to two opposite forces, which can cause magnetic pressure that assists either cell division or cell compression. It is unknown how large this pressure should be to trigger cell division. In the literature, data on this subject are rather sparse. It was demonstrated that a pressure of 100 Pa can drive HeLa cell mitosis^60^. This pressure is an achievable magnetic pressure, e.g., in one of the HGMF systems listed in Table 1.

### Tumor arrest by magnetic pressure

**Figure 5:**
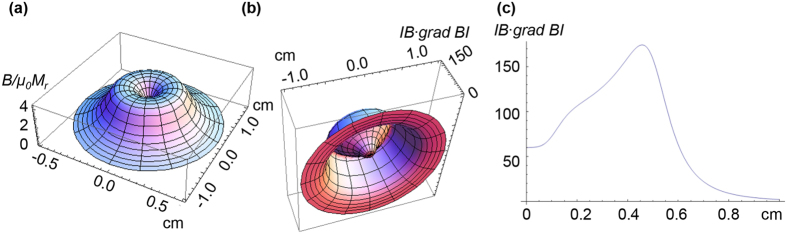
Distributions of the scaled moduli of the magnetic induction (**a**) and magnetic gradient force (**b**) in the plane above a cylindrical magnet with an axial hole. (**c**) 2D-plot of the magnetic gradient force as a function of the radial coordinate. The magnetic induction modulus is normalized to *(μ*_*0*_*M*_*r*_*/4π)*, whereas the modulus of magnetic gradient force is normalized to *R*^*−*^*(μ*_*0*_*M*_*r*_*/4π)*^2^. The calculations were performed for a magnet length 1 cm, magnet radius 0.5 cm, hole radius 0.1 cm, and distance between the magnet top and the plane of calculations of 0.1 cm.

Experiments^61^ suggested that mechanical stress can limit the growth of a spheroid of cancer cells by restricting cell division near the spheroid surface. Here, we show how magnetic pressure can arrest tumor growth. The idea is based on the fact that cancerous cells are enriched by Fe, and therefore they are more paramagnetic than healthy cells^62^. In such a case, magnetic radial pressure can limit tumor growth due to the attractive magnetic gradient force acting on the “paramagnetic” cancerous cells. An example of magnetic field and gradient distributions above cylindrical magnets with a hole is shown in Fig. 5 (details of the calculations can be found in Methods). Magnetic pressure on tumor can be calculated as *P*_*tum*_ = *fw*, where *f* is the force density given by Eq. 2 and *w* is the width of the area corresponding to the maximum of the magnetic field gradient shown in Fig. 5. Estimations of the magnetic pressure on cancerous tissue with magnetic susceptibility χ = 6.3 10^−6^ (in SI units)^62^ for the calculated maximal value of the magnetic gradient, B|∇B|/(*R*^*−1*^*(μ*_*0*_*M*_*r*_/4*π)*^2^) ≈ 160 (see Fig. 5 (b) and (c)) and magnet radius *R* = 5 mm, hole radius 0.1 mm and w = 1 mm, give pressure *P*_*tu*m_ ≈ 1 Pa = 1 pN μm^−2^, which value seems to be not sufficient to affect cell functions. However, |∇B| grows as the hole radius decreases or the distance *z* goes to zero (see Table 1 and Eq. 13 in Methods). Thus, adjusting the hole radius and distance, the magnetic gradient can be increased by hundreds of times to achieve pressures of hundreds of pascals, which can prevent cells from dividing. For example, it was shown in ref 61 that an external osmotic pressure as weak as 500 Pa slowed the growth rate of a tumor spheroid.

## Discussion

**Figure 6:**
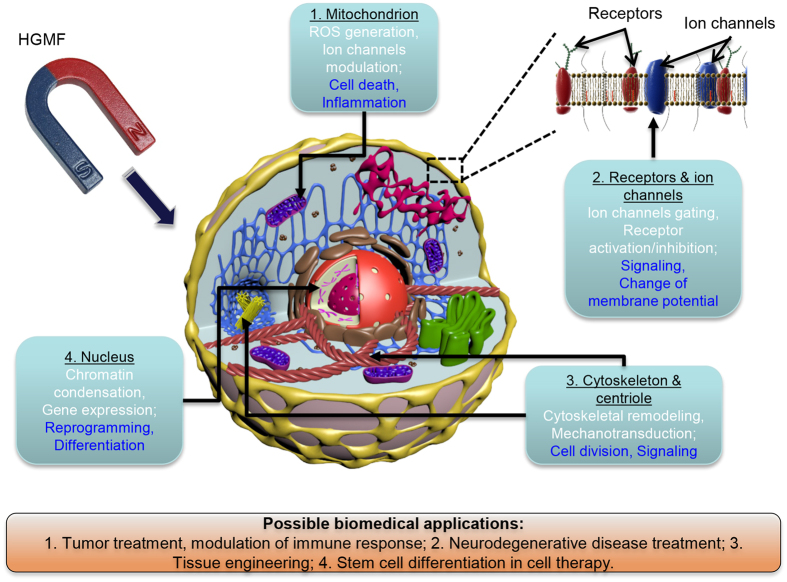
Schematic illustration of the possible applications of HGMFs and intracellular effectors.

By summarizing the analyses of the above-considered phenomena, models and suggested mechanisms, one can identify the following intracellular effectors of applied HGMFs. We use the term “effector” to indicate a structural component of a cell that responds to an applied high-gradient, static magnetic field. Thus, the following are intracellular effectors of an HGMF: i) cytoskeleton remodeling, ii) changing the probability of ion channel on/off switching events, iii) causing the mechanical stress in the membrane, iv) membrane bending, v) migrating membrane receptor proteins, and vi) changing the ion flux balance and membrane potential due to magnetic gradient forces. A schematic illustration of the possible applications of HGMFs and intracellular effectors is shown in Fig. 6. Working alone, each of these effectors can significantly affect cell functions. However, they are not independent and can work in a certain pathway to alter the molecular machinery of a cell and synergize its response to an HGMF. For example, depending on cell type, state and edge, an externally applied HGMF can stimulate cell division, cause cell swelling followed by membrane blebbing and apoptosis, and change the differentiation pathway of stem cells and gene expression. For these and other effects of HGMFs, the magnetic gradient thresholds are shown in Table 2. The cell responses listed in Table 2 do not occur immediately upon application of the HGMF but can be delayed in time. After applying an HGMF, the cell response arises at timescales varying from a fraction of a second to days, which depends on cell type, magnetic gradient magnitude and time of exposure (see Methods).

Magnetic systems generating magnetic fields with gradients on the order of 10^9^Tm^−1^ would allow for significant alteration of the membrane potential in accordance with predictions based on Eq. 1. Changes in membrane potential have proven to be pivotal not only in normal cell cycle progression but also in malignant transformation. Thus, driving the membrane potential with HGMFs opens new opportunities to study intercellular and intracellular processes and provides new routes to controlling cell fate. By understanding the ways in which HGMFs can be utilized to selectively generate the required cellular responses, we can begin to consider magnetic fields as tiny non-invasive tools that can remotely alter the cell machinery, promising broad application potential in cell therapy, neurobiology and nanomedicine. Ultimately, to address the most demanding challenges in medicine utilizing magnetic fields, it is necessary to answer the question: what are the parameters that can reliably allow us to define magnetic field effectors and cause-effect relationships between magnetic field application and cell response? To a large extent, by achieving experimental facilities that provide the highest values of magnetic field gradient, one can expect the discovery of new, exciting, biological effects of magnetic fields.

## Methods

### Generalized Nernst equation for membrane potential

Let us consider the Nernst equilibrium potential in the presence of a high-gradient magnetic field. In equilibrium, without a magnetic field, the free-energy change for the diffusion of an electrolyte into the cell is^63^


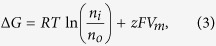


where *z* is the ion valence (z = +1 for a positive, univalent ion), *F* is the Faraday constant, *R* is the gas constant, *T* is the absolute temperature, *V*_*m*_ is the potential difference between the two membrane sides, and *n*_*o*_ and *n*_*i*_ are the ion concentrations outside and inside a cell. By setting ΔG to zero, which is the case when the movement of the ions is at equilibrium, one can arrive at the Nernst equation


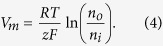


When a high-gradient magnetic field is applied to a cell in medium, the magnetic gradient force acts on ions and can either assists or oppose ion movement through the membrane. The magnetic gradient force is given by


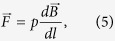


where *p* is the magnetic dipole moment of the ion, ***B*** is the magnetic induction, and the derivative is taken with respect to direction ***l***, which is parallel to the magnetic dipole moment of an ion, ***l***//***p***. Bearing in mind Eq. 5, in this case, when the ions diffuse in the presence of an HGMF, the free energy change is


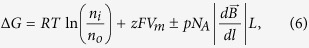


where *L* is the half-cell size and *N*_*A*_ is the Avogadro constant. In Eq. 6, the last term represents the work of the magnetic gradient forces when a mole of magnetic ions diffuses across a membrane; the signs “plus” and “minus” correspond to the two limiting cases: the magnetic gradient force either assists or opposes the electric force exerted on ions moving across the membrane. In equilibrium ΔG = 0, and from Eq. 6, one can arrive at


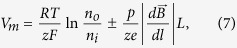


where *e* is the electron charge, which is Eq. 1 (see Results).

### Thermal fluctuation forces

Cell works in a noisy environment created by thermal fluctuations. Therefore, the cellular cytoskeleton exhibits continual fluctuations due to thermal agitation. The thermal fluctuation forces of actin filaments are given by *F*_*th*_ = (*kk*_*B*_*T*)^1/2^, where *k* is the spring constant of a single F-actin filament and the thermal fluctuation energy is *k*_*B*_*T* = 4.1 pN·nm at room temperature. In ref. 64, the effective spring constant for an F-actin network was k_eff_ = 10^−5^ Nm^−1^. Thus, the estimated value of the thermal fluctuation force is *F*_*th*_ = 0.2 pN. This value is slightly less than the measured minimal forces (0.3–0.5 pN) generated by actin filament polymerization^65^.

### Estimation of the electrostatic energy stored in the membrane

For a spherical cell, the electrostatic energy can be calculated as the energy of a charged capacitor


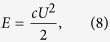


where *c* is the electric capacitance and *U* is the voltage. For a spherical cell membrane with internal and external radii *a* and *b*, respectively, the electric capacitance is


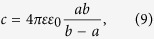


where ε_0_ is the permittivity of free space and ε is the dielectric constant of the lipid bilayer, which typically varies in the range 1–20. By inserting Eq. 9 into Eq. 8, we obtain the electrostatic cell energy as


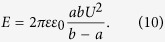


Finally, by inserting the following parameters into Eq. 10: ε = 5, *U* = 70 mV, *a* ≈ *b* = 10 μm and *b* − *a = *5 nm (which is the membrane thickness), one can obtain *E* ≈ 2.7 10^−14^ J.

### Finding strength in the smallest magnets: magnetic systems generating HGMFs

Micro- and nano-magnets are extensively used for a wide spectrum of biomedical applications^66^,^67^. Here, we describe micro- and nano- magnets that can achieve extremely high field gradients. One way to achieve high values of magnetic gradient is to use small magnets and/or to operate near the magnet edges. This idea is based on the fact that the magnetic gradient forces benefits greatly from scale reduction; therefore, micro- and nanomagnets exhibit large magnetic gradient forces. Indeed, it can be easily demonstrated analytically that when all dimensions of a permanent magnet are reduced by the same factor *k* (with all of the magnetic characteristics preserved), the field gradient is multiplied by the reduction factor *k*^68^.

### Magnetized slabs

The magnetic stray field around a uniformly magnetized slab was calculated elsewhere^55^,^59^,^69^,^70^. Near the edge of a long, uniformly magnetized slab of width 2*a*, the magnetic field gradient obeys^71^


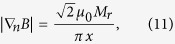


where *x* is the distance to the slab edge, ***n*** is an arbitrary unit vector directed from the slab edge to the point where the field gradient is calculated, and *M*_*r*_ is the remanent magnetization. Eq. 11 is valid for *x«a*, and the modulus of the magnetic field gradient does not depend on the direction of vector ***n***. It follows from Eq. 11 that when approaching the slab edge (*x* → 0), the magnetic field gradient grows and has a singularity. From Eq. 11, estimation with the value of the remanent magnetization of an NdFeB magnet and *x* = 1 μm gives a high value of magnetic field gradient of 5.4∙ 10^5^ Tm^−1^. Similar values of magnetic gradient were measured close to the surface of micro-magnets in ref. 72.

### Axially magnetized cylinder with a hole

We now consider a cylindrical magnet with an axial hole of radius *r*. The magnetic field and its gradient distributions can be calculated with the help of explicit formulas, Eqs 16 and 17 given below. In the limiting case, when *r*→0, directly above the hole, the axial component of the magnetic induction logarithmically depends on the distance, z, from the magnet top along the magnet axis^71^


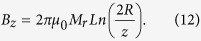


The axial component of the field gradient is


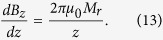


Similarly, for a single, uniformly magnetized, parabolic-shaped magnetic pole used in magnetic tweezers, the maximum magnetic field is given by^73^


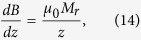


where z is the distance from the magnet pole. Thus, in all of the considered cases, the value of the magnetic gradient increases dramatically when approaching the magnet edge. For example, for a single, parabolic-shaped magnetic pole of size 1 μm, the gradient can reach 3 · 10^6^ Tm^−1^ 100 nm from the tip^73^.

### Magnetic nanoparticles

Let us consider a magnetic nanoparticle with a magnetic moment *p = M*_*s*_*V* (where *M*_*s*_ and *V* are the saturation magnetization and MNP volume). We can represent a nanoparticle as a small, spherical magnet with diameter equal to *2 R*, that is, the single domain MNP acts as a dipole with magnetic moment *p*. Magnetic induction and its gradient at the axis parallel to the magnetic moment direction are given by


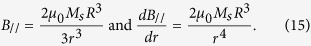


Near the surface of the MNP, at *r = R*, the modulus of the radial magnetic gradient is 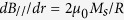, as follows from (15). The perpendicular component, B_⊥_, is two times smaller than B_//_. Thus, for the considered magnet geometry, close to the magnet surface (edge), the magnetic gradient is the same order of magnitude: 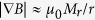, where *r* is the characteristic length scale of the task. We have analytically examined magnetic systems for producing high-gradient magnetic fields and calculated the magnetic flux and gradient distributions that might enable control of the cell shape and functions. The magnetic systems capable of generating HGMFs and formulas allowing rapid estimation of the magnetic field gradient are summarized in Table 1.

### Magnetic field distribution near a cylindrical magnet with an axial hole

The magnetic field and force distributions were calculated with the help of the explicit analytical expressions for magnetic field induction generated by a cylindrical permanent magnet, magnetized along its symmetry axis. For homogeneously magnetized cylinder of the radius, *a* and length *L*, the axial (*B*_*z*_) and radial (*B*_*ρ*_) components of the magnetic field induction can be calculated as^74^:


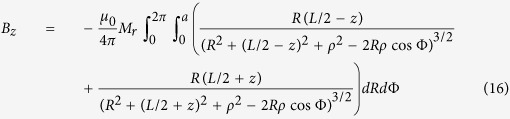


and


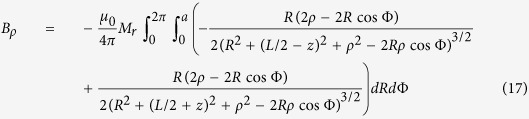


where Φ is the azimuthal angle, *z* is the coordinate along the symmetry axis of a cylinder, *ρ* is the radial coordinate, *M*_*r*_ is the remanent magnetization and *μ*_*0*_ is the permeability of free space. To calculte the magnetic field of a magnet with the axial hole of raddius, *r* one should make the field superposition of two “up-” and “down-” magnetized cylinders: *B*_*z*_* = B*_*z1*_(*a*) *− B*_*z2*_(*r*) and *B*_*ρ*_* = B*_*ρ1*_(*a*) *− B*_*ρ2*_*(r)*, where the subscripts 1 and 2 stand for up-magnetized and down-magnerized cylinders of the radii *a* and *r*, respectivelly.

### Magnetic pressure in the vicinity of magnetic nanoparticles

From Eq. 2, with the help of Eq. 15, one can calculate magnetic pressure as


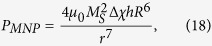


where Δχ is the difference of the magnetic susceptibilities of the lipid membrane and the cytosol.

### Timescales of cell response to HGMFs

The HGMF-induced biological effects mediated by intracellular mechanical stress do not arise immediately upon applying the field. A time delay in cell response to switching on HGMF occurs. In low and moderate magnetic fields, the time delay of the cell response is dependent on the magnitude of the magnetic field gradient but not on the strength of the magnetic field. The following illustrates the hierarchy of the timescales of the observed cell responses to HGMFs for different magnetic gradients. In HGMFs with magnetic gradient of approximately |∇B| ≈ 10^9^ Tm^−1^, a cell response (change of the resting membrane) is expected within a second. Migration and adhesion of stem cells to the edges of micromagnets (at the edge |∇B| ≈ 10^6^ Tm^−1^) with subsequent cytoskeleton remodeling and changes of cell shape were observed during the first 4 hours after cell culture deposition on the magnetic system^46^. During the following 3 days, the cells migrated and occupied the tops of the micromagnets, creating patterns that reflect the spatial distribution of magnetic gradient forces generated by micromagnet arrays^46^. Exposure of the monocytic leukemia cells to a high-gradient magnetic field (up to |∇B| ≈ 10^3^ Tm^−1^) for 24 h induced cell swelling and triggered apoptosis^32^. Changes in DNA organization, gene expression and the differentiation pathway of stem cells were detected after exposure to low-frequency (4 Hz) HGMF with |∇B| ≈ 10^2^ Tm^−1^ for 5 days.

## Additional Information

**How to cite this article**: Zablotskii, V. *et al*. How a High-Gradient Magnetic Field Could Affect Cell Life. *Sci. Rep*. **6**, 37407; doi: 10.1038/srep37407 (2016).

**Publisher's note**: Springer Nature remains neutral with regard to jurisdictional claims in published maps and institutional affiliations.

## Figures and Tables

**Table 1:** Magnetic systems generating HGMFs.

System geometry	Formula for estimation of the magnetic field gradient	Notes	Calculated field and gradient distributions (figures)
Spherical magnetic nanoparticle	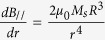	R is radius of MNP	Fig. 3
Two pole to pole faced slabs	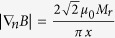 71	*x* is the distance to the slab edge	Fig. 4
Cylinder with a hole	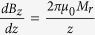 71	The limiting case, when *r*→0; z is the distance from the magnet top.	Fig. 5
Array of micro-magnets	No analytical expression	−	Figs 1 and 2
Parabolic shaped magnetic pole	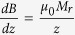 73	Maximum attainable gradient for an optimal diameter.	

To estimate a magnetic field gradient value, use the appropriate equation for a given distance (in meters), substitute the magnet characteristic μ_0_M_r_ (e.g. for a NdFeB magnet μ_0_M_r_ ≈ 1–1.2 T), and then calculate the field gradient.

**Table 2:** Thresholds for the effects of static HGMF.

Effects	Threshold	Cell type	References
Diffusion of ions and biologically-relevant molecules in solutions	∇B^2^ ≈ 10^5^ T^2^m^−1^ to affect the diffusion of paramagnetic molecules FeCl_3_, 0_2_ and plasma proteins.	n/a	39
Magnetically assisted cell migration and positioning	(10^5^–10^6^) Tm^−1^	mesenchymal stem cells	46
Change membrane potential (generalized Nernst equation, Eq. 1)	(10^8^–10^9^) Tm^−1^	all	this work
Local change of membrane potential	(10^8^–10^9^) Tm^−1^	cells with MNPs on membrane	this work
Changing probability of channel switch on/off events	10^3^ Tm^−1^	cells with mechanosensitive ion channels	32
Tumor arrest	(10^4^–10^5^) Tm^−1^	cancer cells enriched by Fe	this work
Magnetically assisted cell division	(10^3^–10^5^) Tm^−1^	HeLa cells, other cancerous cells with low membrane tension	58 and this work
Change differentiation pathway and gene expression	10^2^ Tm^−1^	Mesenchymal stem cells	49
Magnetically assisted endocytosis	(10^2^–10^3^) Tm^−1^	PC-3 cells and fibroblasts	75
Cell swelling	10^3^ Tm^−1^	THP-1 monocytic leukemia cells	32
